# Role enactment of facilitation in primary care – a qualitative study

**DOI:** 10.1186/s12913-017-2537-0

**Published:** 2017-08-23

**Authors:** Tina Drud Due, Thorkil Thorsen, Frans Boch Waldorff, Marius Brostrøm Kousgaard

**Affiliations:** 1 The Research Unit for General Practice and Section of General Practice, Department of Public Health, University of Copenhagen, Copenhagen, Denmark; 2 Research Unit for General Practice, Institute of Public Health, University of Southern Denmark, Odense, Denmark

**Keywords:** Facilitation, Facilitators, Outreach visits, Primary care, Qualitative study, General practice

## Abstract

**Background:**

Facilitation is a widely used implementation method in quality improvement. Reviews reveal a variety of understandings of facilitation and facilitator roles. Research suggests that facilitation interventions should be flexible and tailored to the needs and circumstances of the receiving organisations. The complexity of the facilitation field and diversity of potential facilitator roles fosters a need to investigate in detail how facilitation is enacted. Hence, the purpose of this study was to explore the enactment of external peer facilitation in general practice in order to create a stronger basis for discussing and refining facilitation as an implementation method.

**Methods:**

The facilitation intervention under study was conducted in general practice in the Capital Region of Denmark in order to support an overall strategy for implementing chronic disease management programmes. We observed 30 facilitation visits in 13 practice settings and had interviews and focus groups with facilitators. We applied an explorative approach in data collection and analysis, and conducted an inductive thematic analysis.

**Results:**

The facilitators mainly enacted four facilitator roles: teacher, super user, peer and process manager. Thus, apart from trying to keep the process structured and focused the facilitators were engaged in didactic presentations and hands-on learning as they tried to pass on factual information and experienced based knowledge as well as their own enthusiasm towards implementing practice changes. While occasional challenges were observed with enacting these roles, more importantly we found that a coaching based role which was also envisioned in the intervention design was only sparsely enacted meaning that the facilitators did not enable substantial internal group discussions during their facilitation visits.

**Conclusion:**

Facilitation is a complex phenomenon both conceptually and in practice. This study complements existing research by showing how facilitation can be enacted in various ways and by suggesting that some facilitator roles are more likely to be enacted than others, depending on the context and intervention design and the professional background of the facilitators. This complexity requires caution when comparing and evaluating facilitation studies and highlights a need for precision and clarity about goals, roles, and competences when designing, conducting, and reporting facilitation interventions.

## Background

Facilitation has become a widely used method for implementing quality improvements in health care [1–9]. In primary care facilitation involves an external facilitator, often with a health care background, who visits the practice in order to support a process of change [1, 8]. A recent meta-analysis reported that practice facilitation has a moderately robust effect on the uptake of guidelines in primary care settings [1]. However, the literature on facilitation interventions is marked by substantial variations in design with regard to a) the object of implementation (from relatively simple guidelines to more complex guidelines for chronic care and/or organisational development), b) intensity (duration and number of visits), c) the professional background of the facilitators, and d) their pre-defined roles and tasks. Thus, reviews of the literature have identified a variety of understandings of facilitation and of potential facilitator roles [3, 4, 9].

A continuum has been proposed which conceptualises facilitation as ranging from a goal and task oriented approach to a more holistic approach focusing on organisational development in a broader sense [4, 10–12]. In the goal oriented approach, clinical units are assisted by a facilitator who supports goal setting, provides factual knowledge (e.g. about guidelines), diffuses ideas between settings, and provides project management and technical expertise. In the holistic approach, the facilitator supports a more transformative and empowering change process based on internal discussions, critical reflection, and interpersonal relations [4]. Most interventions are perceived to encompass aspects of both approaches which suggest that facilitators are not necessarily fixed at one point in the continuum, but should be able to move along it depending on the situation [4]. In line with this thinking, subsequent contributions underline the importance of facilitation interventions being flexible and tailored, meaning that facilitation approaches and tools are accommodated to the particular needs, skills and circumstances of the receiving organisations [13, 14].

Hence, the field of facilitation is complex. Although there are common features in definitions and intended activities across studies, these activities are often superficially described. It is rarely explicit how the various facilitation activities are supposed to be or actually are conducted, nor is it stated what the preferred and actual balance is between different activities and facilitation approaches. This entails a profound possibility for variation between intended and enacted facilitation both within and across studies. Further, since each of these activities can be associated with particular competences, the diversity of potential facilitator roles appears to place heavy demands on the competence span of facilitators. Combined with the lack of a clear and consistent operational definition of facilitation [2, 5, 7] this diversity of potential roles opens up the question of how facilitation is actually enacted in specific interventions where a broad understanding of facilitation is adopted. This line of enquiry also fits well with previous calls for more qualitative research aimed at improving our understanding of facilitation as an implementation approach [13, 15, 16].

Prior qualitative studies of facilitation have mostly relied on interviews with facilitators or practices [2, 7, 13, 15, 17–27]. Observations and audio recordings of facilitation visits as a method is less common [15, 16, 19, 20, 23, 25–29]. While such studies have generated new knowledge of practiced facilitator roles and facilitation activities many descriptions in the literature are still fairly broad and undetailed. Hence, there is a need for more detailed knowledge of facilitation enactment e.g. how activities are performed and the balance between them. Further, combining the use of observations and interviews may ensure a more nuanced understanding of how facilitation is enacted.

On this background, the aim of the present study was to explore the enactment of facilitation into specific facilitator roles during facilitation visits. The case used in this study was a facilitation intervention conducted in Danish general practice using peer facilitators. By contributing to a more differentiated and detailed picture of facilitation, we wanted to create inputs to further conceptual discussions and to point to potential areas for improving facilitation as an implementation method.

### The setting of the intervention

Danish health care is mainly tax financed with free-of-charge access to general practice and public hospital services. General practitioners (GP) are private entrepreneurs mostly financed through the tax financed health care reimbursement scheme. Services provided by general practice are regulated by collective agreements between the Danish Regions and the Organisation of General Practitioners [30, 31]. Danish general practice is divided by 45 % partnership practices (co-owned by 2–4 GPs) and 55% solo-practices (of whom some collaborate and share facilities or practice staff) [32]. Practice staff consists of secretaries and nurses, and nurses increasingly conduct selected chronic care consultations (only GPs can perform annual chronic disease check-ups).

### The disease management programmes

Chronic disease management programmes based on the Chronic Care Model [33, 34] have been developed in all five regions of Denmark [35]. As guidelines these programmes outline evidence based treatment and a systematic approach to chronic care including a division of tasks between GPs, hospitals and municipalities for a given chronic disease. The programmes describe the GP’s role as coordinator of care and a systematic proactive approach with population based patient registration, annual chronic disease check-ups and stratification of patients into three levels by risk of complications, complexity, and state of the disease [36, 37]. Several initiatives have been launched to promote the on-going implementation of chronic disease management programmes and to improve chronic care management e.g. IT solutions, lectures, and inter-sectorial collaborations and coordinators. The facilitation intervention studied in this paper was one of the supportive initiatives in the Capital Region of Denmark.

### The facilitation intervention

The facilitation intervention was carried out from 2011 to 2012 in general practice in the Capital Region of Denmark. The overall goal of the intervention was to support the implementation of chronic disease management programmes for type-2-diabetes and chronic obstructive pulmonary disease (COPD) in general practice. A study on the effectiveness of the intervention has been published elsewhere [38].

The intervention was initiated, developed and implemented by the Capital Region of Denmark and the Regional Unit for Quality Development and Continuing Education in General Practice by a project initiator from each organisation and two project managers hired by the Region for this intervention. As external researchers, we were not part of either the design or the implementation of the intervention.

The available documents about the intervention contained limited information on the intended facilitation. Therefore, we interviewed the two project initiators and the two regional project managers for further insight. Hence the description below is based on both documents and these interviews. The facilitators were 14 GPs who were hired on a consultancy basis. According to the interviews with the project initiators when designing the intervention they had assumed that having peer GP facilitators was critical for increasing the legitimacy of the intervention and gaining access to general practice. The facilitators’ educational programme consisted of a one weekend seminar and 10 three-hour meetings over four months. During this period they were updated on the central elements of the chronic disease management programmes, the Data Capture Module (DCM) (see below), and introduced to various implementation and facilitation tools such as the Plan-Do-Study-Act (PDSA) circle [39] and the brown paper method (a visual display of a process with big post-it notes on the wall, where a practice actively focuses on current and future workflows and division of tasks) [40]. The facilitators also participated in workgroups where they developed additional tools. Throughout the rest of the intervention period, network meetings were held approximately every third month where the facilitators discussed their experiences, had further education, and adjusted some of their tools.

All practices in the region were invited to participate in the intervention, but participation was voluntary. Each participating practice was offered up to three facilitation visits of 1 h each. The visits were free of charge and the practice was compensated for lost income. The whole practice was intended to participate during the facilitation visits and in the change process in-between, and most commonly they did. A facilitation visit was a face-to-face meeting in the GP clinic, where the facilitator and the participants were seated around a table or occasionally in front of a computer. The potential topics to cover in the facilitation visits, outlined in the information material provided to the practices, were:

- Workflow and division of tasks for chronic disease check-ups
- International Classification of Primary Care (ICPC) diagnosis-coding
- Patient stratification
- Leadership and organisation
- Collaboration with municipalities and hospitals
- The role of GPs as coordinators of care
- The DCM: a software program for quality development that may provide GPs with an overview of patients’ conditions and treatments. Patient data is automatically collected from the GPs’ electronic health record system. Soon after the facilitation intervention had been introduced it became mandatory for all practices to sign-up to the DCM within two years.

During the facilitation visits the facilitators were intended to act as catalysts for change by:

1. Providing information to practices about the chronic disease management programmes.
2. Engaging in dialogue with the practices about goals for development. In the information material to the clinics it was also stated that gathering the whole clinic for a facilitation visit was an obvious opportunity to talk about work flows, tasks and common goals.
3. Assisting the practices to define specific objectives for the facilitation visits and to choose suitable means for achieving them.
4. Providing tools and suggesting Continuing Medical Education courses and regional supporting initiatives if needed.
5. Ensuring the structure of the facilitation visits by managing agenda setting and time frames, and after each visit providing the practice with a standardised visit report containing the topics discussed, the goals agreed upon and the task to complete in between visits.

The change process was to be driven by the motivation of the practices and based upon their interests and choices of topic. In the interviews with project initiators and project managers they elaborated on the intended facilitation approach by emphasising multiple facilitator roles and a continuum hereof. They described that in order to support a tailored approach the facilitators were supposed to be flexible and the idea of a continuum of facilitator roles was a central element during the education of the facilitators. This continuum ranged from an expert/teacher role at one end to a coaching role at the other end, with the role of a sparring partner in between. Although the facilitators were not expected to be technical or disease specific experts, they were expected to master most of the continuum and to switch between roles according to the situation. The training material stated that the coaching approach was supposed to generate a *“helping and focused conversation between two (or more) persons, where one by using open and focused questions and neutral formulations, gives the other/others the possibility to formulate problems/challenges and create possible solutions”*.

## Methods

An explorative approach was applied in the data collection and analysis, but we were inspired by the various facilitator roles and activities described in the literature, and an idea of a continuum of roles in both the intervention documents and the literature. This study is based on observations, focus groups and individual interviews. TDD observed 30 facilitation visits in 13 practice settings with one to three visits in each setting; (4 of the 13 were joined facilitation visits with collaborating practices, hence a total of 18 practices). The practices were strategically sampled to ensure variation in geography, practice size, and facilitators [41]. An overview of practices and facilitators is presented in Tables 1 and 2. Extensive notes were written by the researcher during the observations of the facilitation visits and the dialogues were audio recorded. Apart from serving as primary data, the observations of the facilitation visits were also used to qualify the interview guides for individual interviews and focus groups. TDD carried out individual, semi-structured interviews with seven facilitators who took part in the observed facilitation visits and TDD and TT ran two concurrent focus groups with approximately half of all the facilitators in each group. The purpose of the individual interviews was to get an in-depth understanding of the facilitators’ behaviours and perceptions. The focus groups explored similarities and differences in the participants’ views, experiences and behaviours which are potentially more clearly illuminated when the participants have the opportunity to reflect on each other’s statements [41, 42]. The themes in the interview guides are presented in Table 3. TDD also interviewed the observed practices and our findings will be reported in a separate paper.

**Table 1:**
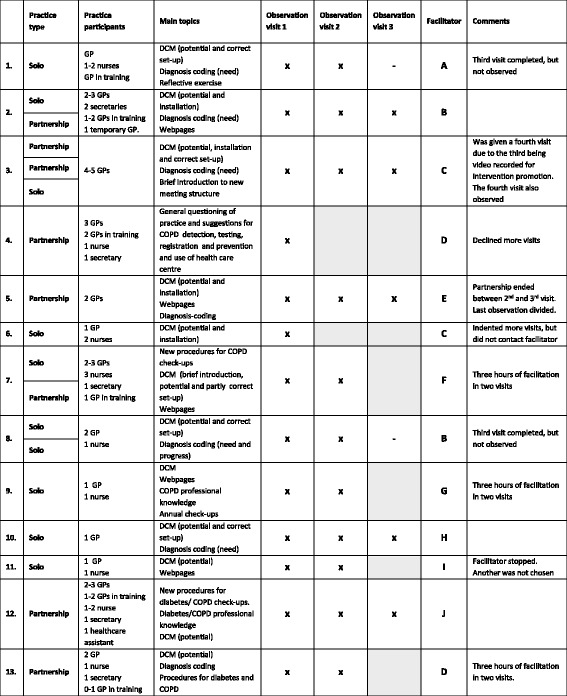
Participating practices (participants, topics and data)

*x is observed visits, - is not observed visits*, shaded areas are not conducted facilitation visits

**Table 2:** Facilitator characteristics and data

Facilitator	Facilitator gender	Facilitator age	Individual interview	Focus group	Observed
A	Female	45–49	+	+	+
B	Female	55–59	+	+	+
C	Male	40–44	+	+	+
D	Male	60–64	+	+	+
E	Female	60–64	-	+	+
F	Female	40–44	+	-	+
G	Female	45–49	+	+	+
H	Female	45–49	+	+	+
I	Female	45–49	-	+	+
J	Female	55–59	-	+	+
K	Male	60–64	-	+	-
L	Male	50–55	-	+	-
M	Female	50–55	-	+	-
N	Male	60–64	-	-	-

+ interviewed, in focus group or observed; - not interviewed, not in focus group or not observed

**Table 3:** Interview guides

Individual interviews
About the observed practice • The preparation of the facilitator and the practice • Their descriptions and experience of the meetings (structure, dialogue, what worked well and what did not) • Their own role at the facilitation visits and in the change process • The practice impact from the facilitation visits • What they perceived as successful/less successful in the specific practice • How the observed facilitation visit differed from other practices and similarities between practices
About their general perception and practice • Their understanding of the facilitator intervention • Their contribution as facilitators • How they were prepared for the role as facilitator • What had influenced their understanding and enactment facilitation • Variations between facilitators and practices • The implication of being GPs • Thoughts about project design (number of facilitation visits, meetings in the practice etc.)
Focus groups
Focus group 1 • Their understanding and description of the facilitator intervention • Their preparation • The content of facilitation visits • Facilitator variations and unity • Their tools • The impact for the practices • The good facilitation visit • Their competences
Focus group 2 • Their understanding of facilitation and the roles of the facilitator • If and why some roles were more often applied • What influenced their roles • Whether they had collaborated on a common understanding • Improvements of their performance over time • Their competences • The significance of them being GPs
Project initiators and project managers
• Background for the intervention • The intervention design • Their understandings of facilitation • The education of the facilitators • Expected changes • Intervention flexibility

All interviews and focus groups were audio recorded and transcribed verbatim after a transcription protocol. We used thematic analysis based on the approach of Braun and Clarke [43] for the interviews and focus groups. TDD performed the initial coding of the data, where data was inductively coded sentence by sentence. The codes were then grouped into themes and sub-themes, which were related to each other and the whole data material and thus refined and connected. We used the NVivo software program in the coding and theme construction process. The notes and audio recordings from the observations where analysed in relation to the themes identified in the interviews. TDD wrote short descriptive narratives of each identified theme including illustrative data extracts. The narratives were read by all authors and discussed during team meetings until consensus on interpretations was reached. This process also lead to a re-reading of data extracts of the different codes and themes, whole interviews and observation notes, and to a re-listening of audio recordings.

TDD, TT and MBK are social scientists (public health, sociology, and political science) and have several years of experience in qualitative health service research. FBW is a GP and professor in general practice. This ensured a good combination of theoretical and methodological competences and brought a number of diverse perspectives into designing the study and analysing the data.

According to Danish law a qualitative study like this one does not require ethical approval by the research ethics committee or written consent by the participants. All participants were promised anonymity and confidentiality.

## Results

According to our observations, facilitation was almost exclusively enacted into four roles during the facilitation visits: the teacher, the super user, the peer and the process manager. These roles were combined in various ways during the facilitation visits with some roles being more pronounced than others. Although there were variations between the facilitators and the facilitation visits (e.g. in the structure and content of the visits and in the balance between roles during the visits) we primarily focused our attention on the similarities in the enactment of facilitation in terms of these roles. Below we present the various roles based on our observations and interviews. Table 4 presents further illustrations of the various roles. At this point we should mention that we found the role of coach described in the intervention design as absent in the observed facilitation visits. Therefore, we also present the reflections of the facilitators on the absence of this role.

**Table 4:** Illustrations of enacted roles

Role	Excerpts from observation notes
Teacher	In practice 3, the facilitator gave a very structured PowerPoint presentation of the DCM. Before beginning, the facilitator said: ‘just interrupt, if anything is unclear’. He then described the system, how to sign-up, how to record and access the patient data, and how to use the system for quality improvement. The facilitator did most of the talking, sometimes answering questions from the practice. The presentation lasted for about one hour with the facilitator loosely skipping over some slides or just reading them aloud.
Super user	In practice 10 with only one GP present, the facilitator emphasised that the GP should be the one sitting in front of the computer. The facilitator sat next to him, guiding him. The GP had installed the DCM some time ago but had not used it. They looked at his ICPC-diagnosis coding percentage and the facilitator showed how him to use the DCM. The facilitator found that the system set-up was not correct and that the GP was not typing all values in the right boxes. The facilitator suggested that the GP contacted his system provider […]. At the next facilitation visit, the facilitator asked the GP if he had increased his coding percentage and once again found problems in the system set-up. The facilitator contacted the IT-system provider who explained how to set up the system and the GP learned this as well.
Peer	In practice 7 the facilitator explained that as an inspiration she would now describe how she had organized the COPD treatment in her own practice. She did so in detail using a PowerPoint presentation. There were a few comments along the way, but mostly the facilitator talked, while the practice was listening. The facilitator underlined that this was her way of organizing the clinic, and that the practice should find out how they wanted to do it. Prior to the facilitation visit, practice 3 had chosen to focus on the DCM. Before giving a detailed introduction to the DCM the facilitator stated *‘there are three main gains from using the DCM and I am not saying it as a representative of the Region, but because I am working with it myself in my practice’.* During the visit several references to the facilitator’s own practice were made, both on the initiative of the facilitator and of the practice.
Process manager	Practice 7 and 12 had chosen to make new procedures for their COPD care. At the end of the first visit the facilitator ensured that 2–3 tasks were specified and that people in charge of each were chosen. At the second facilitation visit, the facilitator began going through the list of tasks asking about the status. In both practices, the appointed people answered that the procedures had been formulated. In practice 7 they were already using the new procedures, and the facilitator asked if they were functioning well, and they agreed that they were. In practice 12 one team member had made a draft and an internal meeting had been scheduled. In neither of the two practices was the content of the procedures discussed.
Coach	As mentioned above Practice 1 was the only observed practice where the facilitator attempted to engage in a more coaching based approach, although this was not fully enacted. The facilitator tried to get the participants to reflect on their own practice through an exercise where each participant wrote down the things that worked well in their diabetes care as well as ideas for improvements and potential barriers. The facilitator then asked each participant about their thoughts. Several issues were brought up during the exercise, but not as a dialogue between the practice members. Rather they stated if they agreed or disagreed with each other’s statements addressing their comments to the facilitator. Also, they did not discuss how to proceed and instead the facilitator suggested that (before the next visit) the practice should arrange an internal meeting to discuss two patient cases and their ideas about how to improve the structure of diabetes care.

### The teacher

In the observed facilitation visits, the facilitators communicated factual knowledge to the practice about central elements of the chronic disease management programmes (such as ICPC coding, the annual chronic disease check-ups, stratification etc.), the DCM, and relevant websites on professional guidelines and municipal health services. The facilitators used more or less structured PowerPoint presentations (shown on PC or projector), speeches, demo versions of the DCM, or demonstrations of relevant websites. In this role the facilitator did most of the talking, but the participants asked questions and commented on the presentations. The presentations on the DCM mainly focused on its potential benefits as the facilitators tried to motivate the practices by providing a rationale for adoption. Practical issues and requirements were often quite randomly provided. Written instructions were generally not provided and notes were not taken by the practice during the facilitation visits.

In the interviews, the facilitators emphasised that disseminating information was less important than having the practice articulate their own ideas and questions. Nevertheless, they also noted that they spent more time teaching than expected:

*To a large extent we do become teachers. You start out with the intention of doing some coaching… but then, when they sit down at the table after a busy day in the clinic, they mostly want some help to get started. And then you often end up teaching. I mean, you have to change between the roles but there is a lot of teaching, I think.* [Facilitator C, focus group]

Several practices had difficulty setting up the DCM correctly in between the facilitation visits and experienced challenges with their IT-system providers. Hence, subsequent visits often focused on these problems concerning the DCM.

### The super user

As a supplement or alternative to didactic presentations, the facilitators often sat down with the GP in front of the clinic computer to provide more practical, hands-on assistance and guidance. For instance, the facilitator would demonstrate unfamiliar features in the patient record system, show how to use the DCM, discover errors in the set-up of the DCM, and talk about DCM data (e.g. coding percentages, missing annual chronic disease check-ups, and improvement thereof at subsequent visits). In this way the facilitators acted as a super user passing on their greater knowledge of the IT systems to less experienced colleagues. The practices were asked to contact their IT-system provider and to set-up the DCM between facilitation visits (a facilitator did this during one visit, because the practice had failed to do so). Although the facilitators could provide some technical assistance, they did not see themselves as technical experts, and according to both interviews and observations they lacked knowledge on the patient record systems that differed from those used in their own practices. Compared to the teaching role, the super user role was more focused on specific practical problems, and the practice participants were more active in terms of asking questions and commenting on the issues at hand. According to the facilitators, the high prevalence of this role in the intervention was due to the concrete and basic needs of the practices, and although used in most practice types the facilitators deemed the super user role highly relevant in smaller practices. The facilitators also perceived that the hands-on approach created a closer relation to the daily tasks of the practice, which was important for the motivation of the participants:

*So in this way things become very hands on like … this makes it more clear to them… when we look up one of their patients in the system and talk about this patient [using the system data] it makes more sense to most of them.* [Facilitator A, individual interview]

### The peer

Most facilitators repeatedly emphasised their professional status as colleagues from general practice. They tended to use the expression “we” in conversation, indicating their common professional identities and working conditions. They emphasised the benefits of increased systematisation in their own clinic (in terms of reduced workload, improved patient care, increased job satisfaction, and better finances). In the interviews, the facilitators often stressed that meaningfulness and ownership were important to ensure change and that they wanted to inspire the practices to change by passing on their own enthusiasm regarding practice development. They often referred to their own experiences from their practice when describing ways of organising the clinic. In some cases such experiences were briefly mentioned during general topic presentations, in other cases the facilitators provided a more comprehensive description of their practice organisation in terms of structure, work division, annual chronic disease check-ups, and use of the DCM. In the interviews the facilitators said that this was meant as inspiration ensuring the practices did not have to *“reinvent the wheel”* during the change process. By referencing their own lack of perfection, their process experiences, their quickly obtained changes, and how they had overcome obstacles, the facilitators aspired to prevent the practices from seeing the change process as overwhelming. It was important for the facilitators that they were not perceived as representatives of the regional health authorities, but as colleagues who knew the business, since this would create a sense of trust and acceptance. Some believed that they could only help the practices because they had been through practice change processes themselves:

*So of course I can use it [own experience]… When I sit there [at the visit] I can say ‘look, we didn’t do any diagnosis coding but then we actually changed and went to almost a 100% in a very short time, so it’s not as difficult and time consuming as it appears.*[Facilitator A, individual interview]

A few facilitators, however, had reservations about using their own practice and procedures as an example, being concerned that it would then become too dependent on the specific facilitator:

*what we have done until now, is to say “well, you can have mine” [instructions of diabetes and COPD care developed in the facilitator’s practice], and I am just thinking is that good enough? ... we are all different, you know, and good at different things, but why should those I visit be saddled with my instruction, who says that it is especially good? I have not until recently realized this, it's not good enough that it is so person-dependent.* [Facilitator H, individual interview]

Additionally, some facilitators emphasised that their position gave them the opportunity to share knowledge and ideas between the visited practices. However, this kind of knowledge sharing was rare in the observed facilitation visits.

### The process manager

It was the responsibility of the facilitators to help clinics with structuring the change process. The facilitators took on this role as process managers in relation to the following aspects:

- *Agenda setting:* Before the first facilitation visit, the clinics were asked to fill out an online questionnaire on their current knowledge and activities in relation to the chronic disease management programmes, and to make suggestions for topics to be addressed at the visit. Nearly all practices filled out the questionnaire, but less than half suggested a topic. At the first visit, the facilitators asked the practices to suggest and choose the overall topics. The extent to which the facilitators guided the choice of topic varied, but they often decided on the more specific content influenced by comments and questions from the practice.

*It’s their project, not my project. So if they are to have ownership, then they must also, and perhaps get inspired, but it must be something they choose to say that they want to work with. [Facilitator F, individual interview]*

*And then, I think everyone should hear about SOFT [webpage listing municipal activities], right? Well. All those things. There are just some things, right, that I think everybody should hear about. [Facilitator A, focus group]*

Further, the practices existing level of knowledge and improvement needs within the overall topic were not always clarified and only the choice of topic was considered; the structure of the visit and the practices’ preferred style of facilitation were not discussed.

b) *Structure:* At the observed facilitation visits there was generally a low degree of structure, several topics and subtopics were covered in varying degrees of detail, either initiated by facilitators or by practices asking questions or telling stories about specific patients. There were variations in the degree of structure, the length of time focused on one topic and whether and how slide presentations were used. The facilitators said that they often had to secure the participation of practice staff. Several times at the observed facilitation visits they asked the staff direct questions or suggested they took charge of particular tasks. However, there were also visits where the dialogue was primarily between the facilitator and the GPs.
c) *Promoting agreement on tasks:* At the first facilitation visits, the facilitators did not attempt to get the practice to set an overall goal although this was a stated intention in the intervention design. Instead the facilitators looked to find agreement on more tangible in-between visit tasks. At some facilitation visits both specific tasks and the people responsible for them were agreed upon; at other visits only the tasks were identified, and sometimes a visit ended abruptly without clearly defining tasks. In most cases the choice of tasks was primarily influenced by the facilitator who suggested the logical next steps. Generally, the facilitator ensured the scheduling of the next visit, but the content was often not explicit. Few facilitators had contact with the practices in between visits.
d) *Follow-up:* In the subsequent facilitation visits the facilitators had the practices do a status report on the previously agreed tasks (e.g. whether procedures had been made, diagnosis coding had improved, or the DCM was used). Hence, the subsequent visits became a deadline and a way of ensuring commitment throughout the process. As one of the facilitators put it:
*It makes it easier when someone comes from the outside… and helps to define goals and tasks. It makes progress easier. Because it makes you think ‘Oh now they come back, now we better start’. So it keeps them at it.* [Facilitator G, individual interview]

However, while status reporting was a means to keep up momentum it rarely fostered further discussions e.g. on implementation of new procedures.

Overall the facilitators tried to manage the facilitation process through agenda setting, task agreement, and follow up. While our observations pointed to occasional problems with these activities the facilitators did not articulate such problems in managing the process. Rather, the challenges they mentioned were not related to their own actions, but to influential contextual conditions (e.g. that larger practices could be more difficult to handle due to lack of time, or that some practices had members that did not attend meetings and delayed or rescheduled meetings).

### The (absent) role of the coach

According to the intervention design, the facilitators were also intended to engage in a coaching approach to help practices to articulate various problems and solutions related to the overall goals of the intervention. The project managers of the intervention also described the coaching approach as one where the facilitator helped to generate internal reflections and discussions between participants at the facilitation visits by asking reflective questions and encouraging dialogue about current and future practice.

However, at the observed facilitation visits, the facilitators did little to stimulate such reflective discussions; rather, they suggested having such discussions in-between the facilitation sessions. Generally, the facilitators tended to do most of the talking at the first visits. At subsequent visits the practices were more actively status reporting, talking about their challenges regarding the DCM, and asking follow up questions. But the internal dialogue between practice members was still very limited. Thus, current organisation was only superficially explored (although GPs and staff answered some questions from the facilitator on current practice), specific change techniques (such as PDSA-circles or brown paper methods) were not used, and there were few discussions of implementation plans and ways of using work division or the DCM for continuous improvement. In only one of the observed facilitation visits (practice 1) did a facilitator attempt to engage in a more coaching based approach with reflections about current practice (Table 4). However, it did not seem fully accomplished, because the level of internal discussion was minimal, and the process was not followed up upon at the subsequent facilitation visit.

In the interviews, the facilitators were asked to reflect on the limited use of the coaching approach. They mainly connected it to the practices having more concrete needs and requesting inspiration from the facilitators’ ways of organising. However according to both interviews and observations, the facilitators did not explicitly clarify the practices’ expected or preferred facilitation approach. A few facilitators commented that they had come into the sessions with the intention of coaching, and momentarily did get into in a reflective mode, but the conversation quickly became more focused on practical problems due to the practices’ needs and expectations. Further, a facilitator reasoned that general practitioners typically are more oriented towards immediate problem-solving of concrete everyday problems than considering their practice and its development on a more reflective and overall level:

*The way that GPs think is very much about handling problems. It’s what we do with patients and this is also how GPs think when they work with [practice] development. That’s why I think it could be interesting to create a more reflective space, to get the thoughts going ‘how are we really doing at the clinic? Is this the clinic we want to be? Are there other areas we should work with to make things more interesting, easier, or better?’ So more general talks and reflections, that is exciting, but this is not the way they are used to think because they work under time pressure and very practically with the patients.* [Facilitator C, individual interview]

Some of the facilitators also indicated that the limited use of the coaching role was because it was less familiar to them, somewhat outside their comfort zone and competences, and linked the use of this role to their personalities. The facilitators also felt that the intervention design had inhibited a more reflective approach, e.g. the facilitation visits took place during the work day which made it difficult for the participant to get into a more reflective mode, and the number of visits was too limited to leave time for more general discussions.

Although we did not observe the coaching role enacted during the facilitation visits, a few of the facilitators said that they did practice aspects of this role in other visits e.g. by using reflective exercises and reflective questioning.

*And then we went through it [their new COPD procedure], so that everyone had like a common basis, and then I started being the annoying one that asked those questions: “what if this or that, then what?.” *[Facilitator H, individual interview]

Further, the facilitators also seemed to vary in their understanding of when a coaching role was enacted. For some it was described as enabling an internal discussion of practice procedures, while others seemed to link coaching to the practice deciding upon the topic, and the facilitator initiating a change process, asking open questions, passing on ideas, and having the practice consider their organisation in between the facilitation visits.

## Discussion

The purpose of this study was to explore the enactment of external peer facilitation in a complex intervention in general practice. We found that facilitation mainly took the form of four different facilitator roles during the sessions: the teacher, the super user, the peer, and the process manager. Thus, facilitation largely took the form of a) didactic presentations and hands-on learning where the facilitators used factual information and experienced based knowledge as well as their own enthusiasm for change and b) process management activities around agreement on tasks and deadlines. While other studies of facilitation have also found such activities to be central to facilitators [7, 13, 15, 17, 44], this study elaborates on the content and balance of the specific roles adopted by the facilitators during interaction, and on the challenges involved in managing several roles. Thus the facilitators sometimes lacked technical knowledge, had problems with structuring the facilitation visits, and did not always manage to ensure a systematic definition of tasks and responsibilities. Perhaps more interestingly we found that the role of the coach envisioned in the intervention design was generally not enacted (in terms of enabling collective reflections and internal discussions at the facilitation visits). This resonates with the study by Rhydderch et al. [16], who found that facilitators had challenges with generating team learning and constructive discussions on practice improvements.

Although the aim of this study was not to assess implementation fidelity as such [45] the limited enactment of the coaching role could be interpreted as a token of limited fidelity. However, it could also be argued that since the enactments of facilitation were often related to both contextual conditions and the stated needs of the participating practices, the enactments were loyal to the intervention’s emphasis on flexibility and tailoring. This suggests that it may be difficult to establish clear fidelity criteria in facilitation interventions with a strong focus on tailoring (and where the nature of facilitation likely renders essential elements difficult to assess).

Several factors influenced the particular enactments of facilitation in this case. First, the professional background of the facilitators shaped their behaviour as they used their professional identity and experiences as GPs to provide knowledge and motivation throughout the process. This is in accordance with studies showing that health professionals bring their professional identity with them into new organisational roles created by various improvement programmes [46, 47]. Further, the facilitators did not have much formal training in the coaching based approach to organizational development which differs significantly from the more familiar activities of intra-professional knowledge exchange familiar to GPs. Second, the context of the intervention probably influenced the enactment of facilitation in a more knowledge based and technical direction (teacher and super user roles) as many facilitation visits came to focus on how to install and use the DCM, which became mandatory for all practices during the intervention period. This entailed profound IT challenges and a need for technical support. If such challenges had not been present, more resources could have been devoted to discussions about how to use patient data in quality development. Third, the design of the intervention (three visits) gave it a relatively low intensity compared to other studies of practice facilitation [1], leaving less room for enacting a more coaching based approach which often requires more time.

Concerning the influence of the facilitators being peer GPs found in this study, it is interesting to note that the current literature reveals that facilitators in primary care most often are nurses or practice assistants, occasionally general practitioners (GPs), and rarely have a background in social science or organisational change [1, 7, 19, 23, 24, 27–29, 48–50]. The influence of the facilitators’ professional background on enactment and outcome would be interesting to investigate in future studies. Currently both studies with peer GP facilitators and with non-peer facilitators have found that practices were satisfied with the facilitation visits [18, 19, 23, 51]. However, the best way to compare the influence of facilitators with different backgrounds would be to study different facilitators within the same interventions, since a comparison between different facilitators in different interventions are impeded by concurrent differences in intervention purpose, content and context. We have only found two studies that compared facilitation visits by a peer GP with facilitation visits by a non-peer within the same intervention. These indicated that facilitation by peer GPs was more effective. However, in both studies the two groups were not completely comparable due to differences between the education given to the facilitators and differences in their prior facilitation experience [17, 52].

In light of the variety of facilitation definitions presented in the literature, the idea of facilitation as a continuum of potential roles to be enacted in a tailored intervention, as well as the findings from this study, it seems appropriate to ask whether the single concept of ‘facilitation’ can meaningfully encompass such a wide range of roles and activities. Thus, although the idea of a continuum of facilitator roles which the facilitator is able to switch between according to the situational needs of the practice is intuitively appealing, it seemed difficult to realise in this intervention. It is important to note that researchers working with a facilitation continuum recently have developed their understanding of facilitation, suggesting that facilitators are categorised according to their experience: novice, experienced, and expert facilitators. In this scheme the novice is not capable of performing all facilitation approaches or roles and needs supervision by more experienced facilitators [5]. This differentiated understanding of facilitation is supported by the findings from this study where most of the facilitators could be perceived as novices, and therefore not yet skilled in mastering a wider range of roles. In the literature of facilitation in health care it is occasionally mentioned which facilitation skills are perceived as essential [16, 25, 44, 53–55]. Nevertheless, the content and length of the facilitators’ education is rarely described in current studies, and knowledge of what their education ought to contain also seems to be lacking [3, 4]. Even though the facilitators in this intervention felt well-prepared, their educational programme had been was quite brief. This is not unusual in facilitation interventions [15, 27, 51, 56]. Therefore, it is possible that an expanded educational programme and on-going guidance from experienced facilitators could improve facilitators’ skills and range. However, we would add that even very experienced facilitators may not be able to move easily along the continuum since few people will have the knowledge (whether medical or technical), the experience, the process management skills, and the interactive facilitation skills required to do this.

The findings of this study regarding enacted facilitation roles can influence future research and intervention designs in various ways. First, the study demonstrates that even within the same intervention facilitation can take on several and diverse forms. Therefore, in order to create a better basis for comparing facilitation interventions and for synthesising findings and the potentials of facilitation as a change and implementation tool, there is a need for conceptual clarification, and for future intervention studies to provide more detailed descriptions of how facilitation is actually enacted. Second, the typology of enacted facilitator roles generated in this study could be taken into consideration when developing programme theories [57] of future facilitation interventions by giving rise to such questions as: What facilitator role(s) will be particularly needed to achieve the intended outcomes given the specific circumstances and what are the expected change mechanisms connecting these? What extra training is required to perform these roles compared with the existing skills of the facilitators? Which resources are needed to accomplish this training? Are certain professions better suited for the enactment of the intended facilitation than others? Third, if a facilitation intervention is monitored or evaluated formatively, the facilitator roles in need of improvement should be identified and given special attention (e.g. through additional training) in a concomitant endeavour to improve change mechanisms and outcomes.

### Strengths and limitations of the study

The facilitation intervention explored in this study framed a variety of facilitator roles and employed a relatively large number of facilitators. Therefore, as a case, the intervention was well suited to shed light on the enactment of different facilitator roles and the potential challenges related to broad framings of facilitation. It may be argued that the design of the intervention was somewhat naïve in assuming that the facilitators would manage to easily move along the continuum of facilitator roles based on their brief education. However, the optimal combination of knowledge, time and resources is rarely present when complex ideas are translated into practice in real life settings and as mentioned above the education of the facilitators in these kinds of interventions is often quite brief. It is a strength of the study that the intervention was explored by methodological triangulation using focus groups, individual interviews, and direct observations. Direct observations produce detailed insights into the “black box” of facilitation and serve to counteract the bias generated when relying solely on post-hoc interviews with participants [41]. It is a limitation of the study that we may not have reached data saturation concerning the observations. Thus, at the observed facilitation visits we did not see the coaching role enacted although a few facilitators told of activities related to this role in other facilitation visits. However, our findings on the limited enactment of this role were generally supported by the interviews and focus groups.

## Conclusion

In this study of facilitation in the context of implementing chronic disease management programmes in general practice, facilitation was enacted through different facilitator roles. The facilitators engaged in various forms of factual and experienced based knowledge transmission using their peer status as a source of inspiration and credibility, and supported the process by ensuring task and subsequent follow-up. They generally did not enact the coaching role defined by the intervention in terms of generating collective reflections on problems and improvements at the facilitation visits. There were also indications of occasional challenges regarding some of the other roles (e.g. limited technical knowledge, limited structure during the visits, tasks not defined).

Previous reviews have established that facilitation is a complex phenomenon in both theory and practice. Our results complement the existing literature by suggesting that facilitation is enacted in various ways and that some facilitator roles are more likely to be enacted than others depending on the design, content, and context of the intervention as well as on the professional background of the facilitators. This complexity calls for caution when comparing results from facilitation studies and points to a critical need for precision and clarity about goals, roles, and competences when designing, conducting, and reporting facilitation interventions.

## Abbreviations

COPD: Chronic obstructive pulmonary disease
DCM: Data capture module
GP: General practitioner
ICPC diagnosis coding: International classification of primary care diagnosis coding
